# Effects of Methyl Salicylate on Host Plant Acceptance and Feeding by the Aphid *Rhopalosiphum padi*

**DOI:** 10.3389/fpls.2021.710268

**Published:** 2021-08-13

**Authors:** Velemir Ninkovic, Robert Glinwood, Ayse Gül Ünlü, Suresh Ganji, C. Rikard Unelius

**Affiliations:** ^1^Department of Ecology, Swedish University of Agricultural Sciences, Uppsala, Sweden; ^2^Department of Crop Production Ecology, Swedish University of Agricultural Sciences, Uppsala, Sweden; ^3^Department of Chemistry and Biomedical Sciences, Linnaeus University, Kalmar, Sweden

**Keywords:** plant defense, plant resistance, herbivores, plant volatiles, VOCs, olfactory response, semiochemicals, methly salicylic acid

## Abstract

Methyl salicylate (MeSA) is a volatile shown to act as an inducer of plant defense against pathogens and certain herbivores, particularly aphids. It has been shown to have potential for aphid pest management, but knowledge on its mode of action is lacking, particularly induced plant-mediated effects. This study investigated the effects of exposing plants to MeSA on the host searching, host acceptance and feeding behavior of the bird cherry-oat aphid *Rhopalosiphum padi*. Barley plants were exposed to volatile MeSA for 24 h, after which biological effects were tested immediately after the exposure (Day 0), and then 1, 3 and 5 days after the end of the exposure. Aphid settling on MeSA-exposed plants was significantly reduced on days 0, 1 and 3, but not on day 5. In olfactometer tests, aphids preferred the odor of unexposed plants on days 1 and 3, but not on day 0 or 5. Analysis of volatiles from exposed and unexposed plants showed higher levels of MeSA from exposed plants, most likely absorbed and re-released from plant surfaces, but also specific changes in other plant volatiles on days 0, 1 and 3. High doses of MeSA did not affect aphid orientation in an olfactometer, but lower doses were repellent. Analysis of aphid feeding by Electronic penetration graph (EPG) showed that MeSA exposure resulted in resistance factors in barley plants, including surface factors and induced systemic factors in other tissues including the phloem. The results support the potential of MeSA as a potential tool for management of aphid pests.

## Introduction

Volatile semiochemicals are increasingly being considered as promising components of integrated management strategies against insect pests (Smart et al., 2014). Semiochemicals may include compounds that directly repel pests, attract natural enemies, or they may be “elicitors” that induce defensive pathways that confer resistance in the host plant (Maffei et al., 2012). Certain semiochemicals may potentially have more than one of these modes of action. The salicylic acid biochemical pathway in plants, mainly activated in response to the attack of sap feeders, is generally considered to provide defense against biotrophic pathogens, but it also appears to function against certain herbivorous arthropods, particularly those with a piercing/sucking feeding mode (Aerts et al., 2021). Methyl salicylate (MeSA) is a volatile compound associated with the salicylic acid pathway in plants. MeSA has been shown to act as a mobile signal for systemic acquired resistance (SAR) by being converted to salicylic acid (Park et al., 2007) and is known to promote the expression of defense related genes in response to herbivores and pathogens (Li et al., 2002).

MeSA may have different modes of action that can be exploited for pest management (James, 2003; Ninkovic et al., 2003; Byers et al., 2021). It is attractive to a range of natural enemies of arthropod pests (Mallinger et al., 2011; Orre Gordon et al., 2013) and possibly birds (Rubene et al., 2019), and has shown repellency against aphids (Glinwood and Pettersson, 2000; Prinsloo et al., 2007; Digilio et al., 2012). MeSA is often reported as a plant volatile that is induced by insect feeding, and may play a role in defense signaling within (Heil and Ton, 2008) or between plants (Shulaev et al., 1997). Thus, the compound can act as a defense-elicitor (Heil and Ton, 2008), providing an additional mode of action against pests.

MeSA may have particular potential against piercing/sucking pests such as aphids. In fact, the capacity of MeSA to suppress aphid populations in crops has been demonstrated in a number of studies (Pettersson et al., 1994; Ninkovic et al., 2003; Prinsloo et al., 2007; Xu et al., 2018). Outside of the attraction of natural enemies, the mode of action of the compound is not always clear, particularly regarding the relative importance of direct and plant-mediated effects. This is particularly true for the bird cherry-oat aphid, *Rhopalosiphum padi* L. which has been successfully managed by releasing MeSA in cereal fields (Pettersson et al., 1994; Ninkovic et al., 2003). *R. padi* alternates between a winter host, *Prunus padus* L., and summer hosts including grasses and cereals. There is evidence that MeSA is involved in this host alternation process, and may act as an aphid feeding-induced cue mediating migration from winter host (Glinwood and Pettersson, 2000; Pickett et al., 2017). Moreover, previous studies have shown that the exposure of cereal plants to MeSA can reduce *R. padi* host acceptance (Ninkovic et al., 2003; Glinwood et al., 2007), suggesting that MeSA could affect this aphid both directly as an ecological cue in the lifecycle, and indirectly by eliciting unfavorable changes in the host plant.

Aphids find, select and colonize host plants through a step-wise process (Pettersson et al., 2007). Initially volatile chemical and/or visual cues mediate attraction. After landing on the plant cues from the plant surface may be involved in the decision to begin feeding. The feeding process is highly developed; the stylet probes the plant tissues and navigates mostly between cells eventually piercing the phloem sieve elements to commence feeding. Protective effects against aphids induced by exposing plants to MeSA could therefore be expressed at several different stages or in different plant tissues. Thus, although MeSA has been shown to suppress *R. padi* populations in crops, it is still unclear how plant-mediated effects, including disruption of feeding, may contribute to this.

*R. padi* is a major pest of cereals in many temperate regions including Sweden (Wiktelius et al., 1990), with increasingly limited options for insecticide treatment. The application of MeSA could be promising in the development of more sustainable approaches to control *R. padi* and other aphid species. Still, the direct effects on plants and plant-mediated effects against aphids need further attention. The aim of this study is therefore to investigate the effect of exposing barley plants to volatile MeSA on the behavior of *R. padi* related to host finding, host acceptance and feeding. We exposed barley plants to volatile methyl salicylate for 24 h, then investigated the dynamics of *R. padi* responses to MeSA exposed plants over a number of days including olfactory responses, host acceptance and feeding behavior. We also analyzed MeSA-induced changes in the volatile profiles of exposed plants.

## Materials and Methods

### Aphids

Bird Cherry-oat aphid, *R. padi*, was reared on oat plants (*Avena sativa* cv. Belinda) in multi-clonal cultures in a greenhouse at 20 ± 2°C with a L16:D8 light cycle. Apterous viviparous aphids of larval instars 2–4 used in the all experiments were collected from cultures immediately prior to bioassays.

### Plants

Barley plants, *Hordeum vulgare L*. (cv. Scandium) were grown in plastic pots (8 × 8 × 6 cm) with soil (Hasselfors Garden Special, Sweden) with one plant/pot (apart from volatile collections where 10 plants/pot were used). Plants were grown in a greenhouse chamber at 20±2°C with 16D:8L light cycle with supplementary lighting from HQIE lamps.

### Chemicals

Commercially available authentic standards and other chemicals were obtained from (Sigma-Aldrich, Sweden). Methyl salicylate triple-deuterated on the methoxy group (D-MeSA), was synthesized by hydrolysis of ethyl salicylate to get salicylic acid. The second step was an acid-catalyzed (HCl) esterification with deuterated methanol (See Supplementary Material for spectroscopic data).

### Plant Exposure to Methyl Salicylate

Barley plants were exposed to volatile MeSA inside a twin-chamber exposure cage system (Ninkovic et al., 2002). The system consisted of a series of clear Perspex cages divided in to two chambers (each chamber 10 × 10 × 40 cm) connected by an opening (Ø 7 cm) in the middle wall. Each Perspex cage was connected to a vacuum tank pump from which the air was removed by a fan and vented outside the greenhouse. Airflow through the cages was 1.3 l/min. MeSA (98%, CAS no. 119-36-8) was purchased from Sigma-Aldrich, Sweden. 10 μl MeSA was applied to a 9 cm filter in a Petri dish placed in the front chamber, and pots with plants were placed in the rear chamber. The mean aerial concentration of MeSA in the exposure cages using this method was measured in a previous study as: first 4 h 300 ng/l (1.9 nM) (peak 625 ng/l, 4.5 nM), following 8 h 50 ng/l (0.3 nM) (Glinwood et al., 2007).

Control plants were treated in the same way except that no MeSA was applied to the filter papers. For each experiment, barely plants at the two-leaf stage (11 days after sowing) were used. Only one plant per pot was grown to avoid pseudo replication and unplanned differences in the cage caused by treatment. Individual pots were watered using an automated drop system (DGT Volmatic) without additional fertilizer. The two-chamber cages were kept in a greenhouse chamber at 20±2°C with 16D:8L light cycle with supplementary lighting from HQIE lamps.

The filter papers with and without MeSA were removed from the inducing chamber after 24 h. After filter papers were removed, plants were used immediately in experiments (Day 0), or kept for 1 day (Day 1), 3 days (Day 3) or 5 days (Day 5) in the responding chambers.

### Aphid Plant Acceptance

The effect of plant exposure to MeSA on aphid plant acceptance was assessed by means of a no-choice settling test (Ninkovic et al., 2002). A 50-ml polystyrene tube was placed over the second leaf of plants at the two-leaf stage. The upper end of the tube was covered with a net and the lower end with a plastic sponge plug with a slit for the leaf. The tube was supported with a stick to avoid mechanical damage to the plant. Ten aphids were placed in the tube and the number of aphids settled (not walking) on the leaf was recorded after 2 h, since this is sufficient time for aphids to settle and reach the phloem with their stylets (Prado and Tjallingii, 1997). The tests were performed on a bench in a separate greenhouse chamber under the same conditions as the MeSA exposures. Twenty plants per treatment were tested.

### Analysis of Aphid Probing Behavior Using Electronic Penetration Graphs

The effect of plant exposure to MeSA on aphid probing and feeding in plant tissues was monitored using the DC-EPG technique (Tjallingii, 1986, 1988). Four plants treated with MeSA and four control plants were placed in a Faraday cage where aphid probing and feeding behavior was recorded simultaneously during 8 h for each experiment. Each pot with one plant was placed in a Petri dish. To prevent interaction between treated and control plants via volatiles, the Faraday cage was divided into two chambers each with independent airflow and ventilation. The placement of treated and control plants was alternated between chambers and between tests.

Aphids were collected using a soft brush and then held in place by a vacuum device to apply a small drop of conductive water-based silver glue on the dorsum to which a thin gold wire (20 μm) electrode about 2 cm long was attached. The other end of this electrode was attached to a 3 cm long copper wire connected to a channel (a first stage amplifier with 1 GΩ input resistance) (Tjallingii, 1988). Each wired insect was placed on the abaxial side of the second leaf of a barely plant, about 1 h after collection from the aphid culture. A second electrode (2 mm in diameter and 10 cm long copper rod) was inserted into the soil of the potted plant and connected to Giga-8 (plant voltage output of EPG device) (Wageningen University, The Netherland). The EPG device was connected to A/D converter at 100 Hz (DATAQ Instruments, USA) and it was in turn connected to a computer where waveform signals of eight plants, divided between two Faraday cage chambers, were recorded using PROBE 3.0 software.

EPG signals were analyzed using STYLET A software (epgsystems. eu). Identification of the waveform patterns was made according to Tjallingii (1990). Aphid probing behavior was divided into five main phases: the non-probing phase (np), the probing phase (C) representing aphid stylet penetration in plant tissues which was further divided into the pathway phases (epidermis first stylet contact waveform A, intercellular sheath salivation waveform B; stylet movements waveform C, and an intracellular stylet puncture waveform pd “potential drop”), the phloem phase (E) divided in “salivation activity” (El) and sap ingestion (E2), stylet penetration difficulties (F) and xylem phase (G) active sucking of water from xylem elements. A number of variables (parameters) most relevant for aphid resistance, comparable to those used by Marchetti et al. (2009), were derived from the EPG signals. EPG variables were expressed in mean numbers, total durations of waveform periods and durations of waveform periods before or after certain events per treatment.

### Collection and Identification of Plant Volatiles

To investigate the effect of plant exposure to MeSA on barley volatile production, volatiles were collected and identified as described below. Pots containing 10 barley plants were exposed to methyl salicylate as described above. Control plants were kept inside two-chamber cages without exposure to the chemical. Volatiles from barley plants were collected by air-entrainment (Glinwood et al., 2011). The whole pot with 10 plants was placed inside a polyester (PET) cooking bag (Stewart-Jones and Poppy, 2006) (60 × 55 cm, Toppits, Melitta, Sweden). A glass liner containing 50 mg of the molecular adsorbent Tenax TA (Atas GL Intl., Veldhoven, Netherlands) was inserted through a small hole cut in one corner of the bag. A positive pressure push–pull system was used, with charcoal-filtered air pushed in through a Teflon tube inserted through a small hole in the bottom of the bag, at 600 ml/min and pulled out over the adsorbent at 400 ml/min. Bags were baked in an oven at 140°C for 2 h immediately prior to the entrainment. Charcoal filters and Tenax tubes were baked at 180 and 220°C respectively under a flow of nitrogen for 16 h. At time points Day 0, Day 1 and Day 3 (after the end of the 24 h exposure to methyl salicylate), pots of plants were removed from exposure cages and volatile collection was started immediately for a total duration of 24 h. For each treatment and time point, six separate pots of plants were entrained. Three collections were made from plastic pots containing only soil and only volatiles appearing in the plant samples but not in the soil controls were quantified.

Volatiles were analyzed by gas chromatography-mass spectrometry (GC/MS) on an Agilent 7890N (Agilent Technologies) GC coupled to an Agilent 5975C mass selective detector (electron impact 70 eV). The GC was equipped with an HP-1 column (100% dimethyl polysiloxane, 50 m, 0.32 mm i.d. and 0.52 μm film thickness, J&W Scientific, USA), and fitted with an Optic 3 thermal desorption system (Atas GL Intl., Veldhoven, Netherlands). The liner containing the Tenax with absorbed volatiles was placed directly into the injector and volatiles were thermally desorbed starting at 30°C/0.5 min, and rising at 30°C/s to 250°C. The GC temperature program was 30°C/4 min, 5°C/min to 150°C/0.1 min, 10°C/min to 250°C/15 min, using helium as carrier with a flow rate of 1.3 ml/min. Volatile compounds were identified by comparison against a commercially available library (NIST 08) and by comparison of mass spectra and retention indices with commercially available authentic standards (Sigma-Aldrich, Sweden). Amounts of compounds in the samples were quantified based on the cumulative abundances for three ions selected with the criteria that they were typical and abundant for the target compound. These were then compared with response curves constructed using commercially available chemical standards (Sigma-Aldrich, Sweden) to estimate the amount present in the sample.

### Estimation of Absorption/Re-release Compared With *DeNovo* Production of MeSA in Exposed Plants

The aim was to estimate the relative contribution to the total MeSA measured in the headspace of exposed plants by absorption/re-release from the leaves compared with *de novo* emission by the plant. Plants were exposed to deuterated-MeSA (D-MeSA) (see Supplementary Material - Synthesis of Deuterated MeSA), using the same method described above.

As described above, D-MeSA was removed from the exposure cage, and pots containing 10 barley plants were used for volatile collection (24 h) immediately (Day 0) or 1 or 3 days after removal of D-MeSA. Volatiles were collected according to the method described above, except that glass collection tubes contained the adsorbent Porapak Q (50 mg, mesh 50/80, Supelco, Bellefonte, PA, USA). These tubes were prepared by rinsing with dichloromethane and baking for 4 h at 140 °C under nitrogen flow. After the volatile collection, collected volatiles were eluted from the absorbent tubes using 500 μl dichloromethane then concentrated to 50 μl under a low flow of nitrogen.

A 2 μL aliquot of the extracted sample was injected into an Agilent 7890A GC (Agilent Technologies, Santa Clara, CA, USA) equipped with a cold-on-column injector and fitted with an HP-1 column (30 m, 0.25 mm i.d., and 0.25 μm film thickness; J &W Scientific, Folsom, CA, USA) coupled to an Agilent 5975C mass selective detector (electron impact 70 eV, 230 °C). The GC program was set to start at 30 °C for 1 min, and set to rise 20 °C/min to 250 °C. The carrier gas was helium with a flow rate of 1 ml/min. The mass selective detector was programed in selected ion monitoring (SIM) mode, with the quantification ions *m/z* =155 for D-MeSA and *m/z* = 152 for MeSA and a confirmation ion *m/z* = 92. Quantifications were made using the abundances of quantification ions in the collected samples compared with those in an injected authentic standard of D-MeSA or MeSA (10 ng).

### Olfactometer Bioassays

The effect of plant exposure to MeSA on aphid responses to plant volatiles was tested using a two way airflow olfactometer (Vucetic et al., 2014). *Three* separate experiments were carried out:

(i) *aphid olfactory response to odor from exposed and unexposed plants*. Plants used as odor sources were kept in two-chamber cages, as described above. A two-chamber cage with a plant previously exposed to MeSA was directly connected to one arm of the olfactometer, and a two-chamber cage with an unexposed plant was connected to the opposite arm.

(ii) *aphid olfactory response to synthetic odor blends*. Using chemical standards, odor blend mixtures were constructed based on the occurrence and proportions of compounds identified by GC/MS in the plant headspace for MeSA-exposed and unexposed plants at Day 1 (1 day after removal of MeSA from the exposure chambers). For each treatment, five blends were constructed over a range of different concentrations: 100×, 10×, 1×, 1/10 and 1/100 the concentration in the headspace collections. Synthetic blends (10 μl of a solution in hexane) of MeSA-exposed and unexposed plants were dosed onto small filter paper squares placed in a tube connected to the arm of the olfactometer. Chemicals were obtained from commercial suppliers: (Z)-3-hexen-1ol (98 % purity, Sigma Aldrich Inc., St. Louis, MO, USA), 6-methyl-5-hepten-one (99 % purity, Sigma Aldrich), myrcene (90 % purity, Fluka, Buchs, Switzerland), (Z)-3-hexen-1-yl acetate (99 % purity, Sigma Aldrich), linalool (97 % purity, mixture of (S) and (R) isomers, Sigma Aldrich), methyl salicylate (98 % purity, Sigma Aldrich), longifolene (99 % purity, ABCR GmbH & Co., Karlsruhe, Germany).

(iii) *aphid olfactory response to individual chemicals differing significantly between profiles of MeSA-exposed and unexposed plants*. The procedure was as for (ii) above using the same concentration range of substances, with hexane lone as control.

Airflow in the olfactometer was 180 ml/min, measured with a flowmeter at the arm inlets. A single aphid was introduced into the olfactometer and, after an adaptation period of 10 min, the aphid's position in the arena was recorded every 3 min over a 30-min period. The accumulated number of visits in the arm zones (excluding the central neutral zone) was regarded as one replicate. Each test was repeated 20 times (aphids) for each treatment. Each individual aphid was used only once. If an aphid was inactive in the olfactometer (i.e. observed to be stationary in the same position for three consecutive observations) it was removed and the bioassay restarted with a fresh aphid. To account for any positional bias the position of treatments in the olfactometer was switched between the left and right arms in each separate olfactometer.

### Statistical Analysis

Statistical differences in aphid settling between treated and control plants were analyzed using t-tests for independent samples. Data for all variables were subjected to tests for homogeneity of variances. As the EPG data were not normally distributed, paired comparison of means of MeSA treatments with controls was done by nonparametric Mann–Whitney *U*-test. Plant volatile data followed the assumptions of normality and were analyzed by one-way analysis of variance (ANOVA) following SQRT transformation to reduce heteroscedasticity when necessary. Aphid olfactory response data were analyzed by Wilcoxon matched pairs tests. All statistical tests were performed with the Statistica software (TIBCO Software Inc., 2018).

## Results

### Aphid Settling on MeSA-Exposed and Unexposed Plants

**Figure 1:**
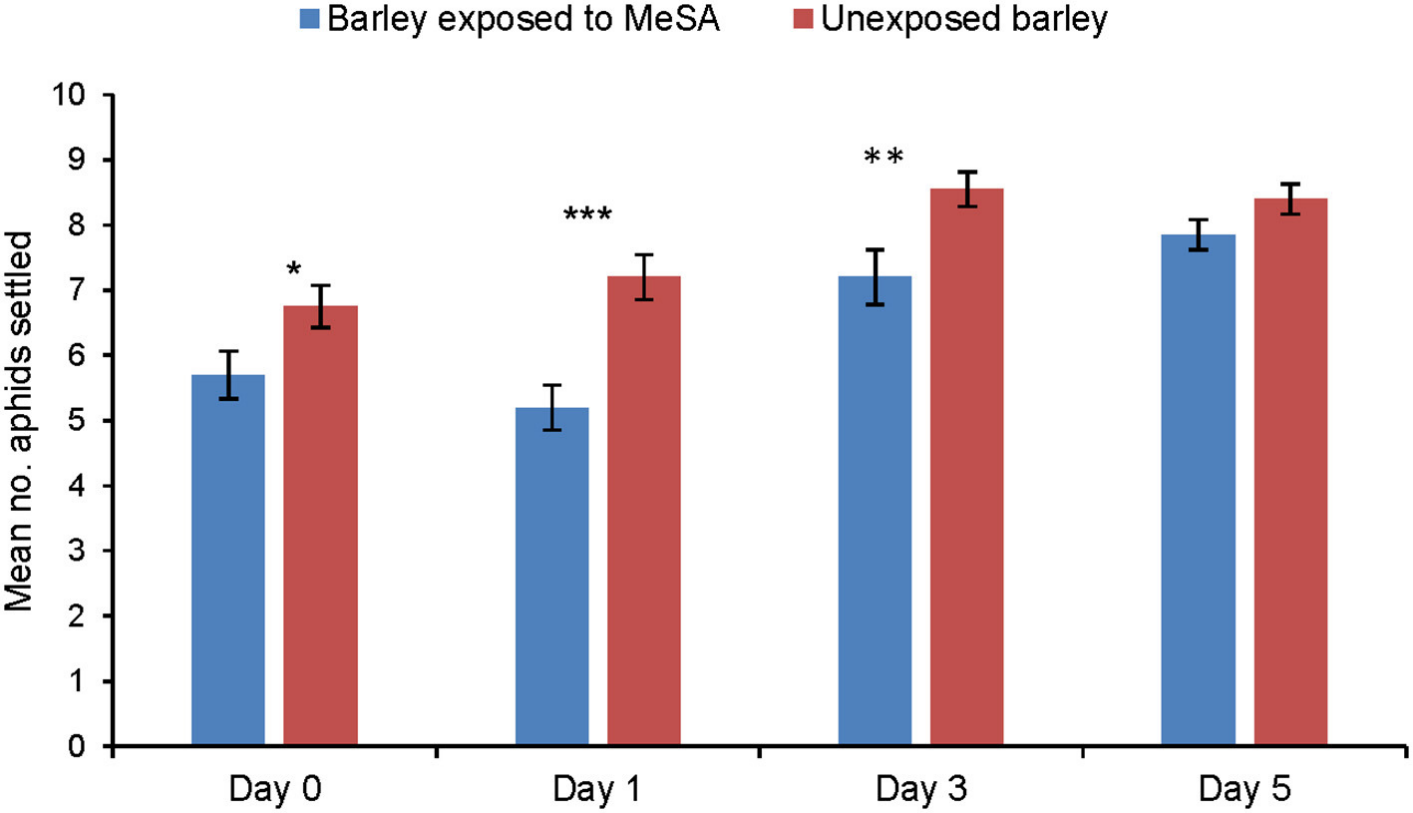
Settling (mean number of aphids settled ± SE) of *R. padi* on unexposed barley plants or on plants exposed to MeSA (directly after removal of MeSA exposure = Day 0 and at time points 1, 3 and 5 days after removal of MeSA exposure). *p ≤ 0.05; **p ≤ 0.01; ***p ≤ 0.001 after t-tests.

Immediately after a 24 h exposure to MeSA (Day 0), aphid settling was significantly reduced on exposed barley plants in comparison to unexposed plants (*p* = 0.037). Aphid settling was also significantly reduced on exposed plants at Day 1 (*p* = 0.0004) and Day 3 (*p* = 0.01) after removing MeSA, before returning to the same level as unexposed plants at Day 5 after removal of MeSA (*p* = 0.104) (Figure 1).

### Aphid Olfactory Response to Odor From MeSA-Exposed and Unexposed Plants

**Figure 2:**
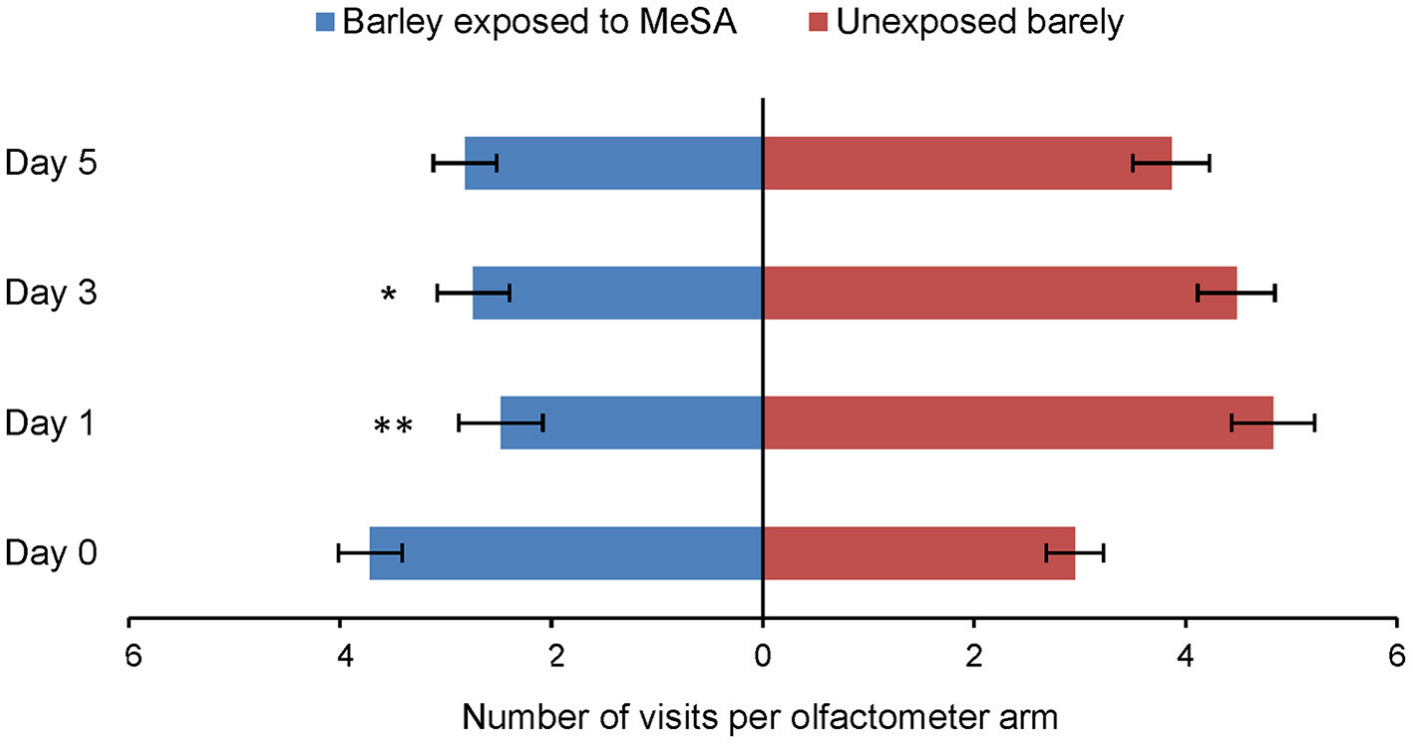
Olfactory response of *R. padi* (mean visits in olfactometer arm ± SE) to odor of unexposed barley plants or to plants exposed to MeSA (directly after removal of MeSA exposure = Day 0 and at time points 1, 3 and 5 days after removal of MeSA exposure). *p ≤ 0.05; **p ≤ 0.01 after Wilcoxon matched pairs tests.

Aphids were observed significantly less often in the olfactometer arm containing odor of barley plants previously exposed to MeSA than in the arm with odor of unexposed plants at Day 1 (Wilcoxon test, *Z* = 2.79, *p* = 0.006) and Day 3 (Wilcoxon test, *Z* = 2.35, *p* = 0.018). These significant reductions in aphid preference to treated plants were not observed at Day 0 (Wilcoxon test, *Z* =1.36, *p* = 0.173) or Day 5 (Wilcoxon test, *Z* = 1.63, *p* = 0.103) (Figure 2).

### Volatile Profiles of MeSA-Exposed and Unexposed Plants

**Figure 3:**
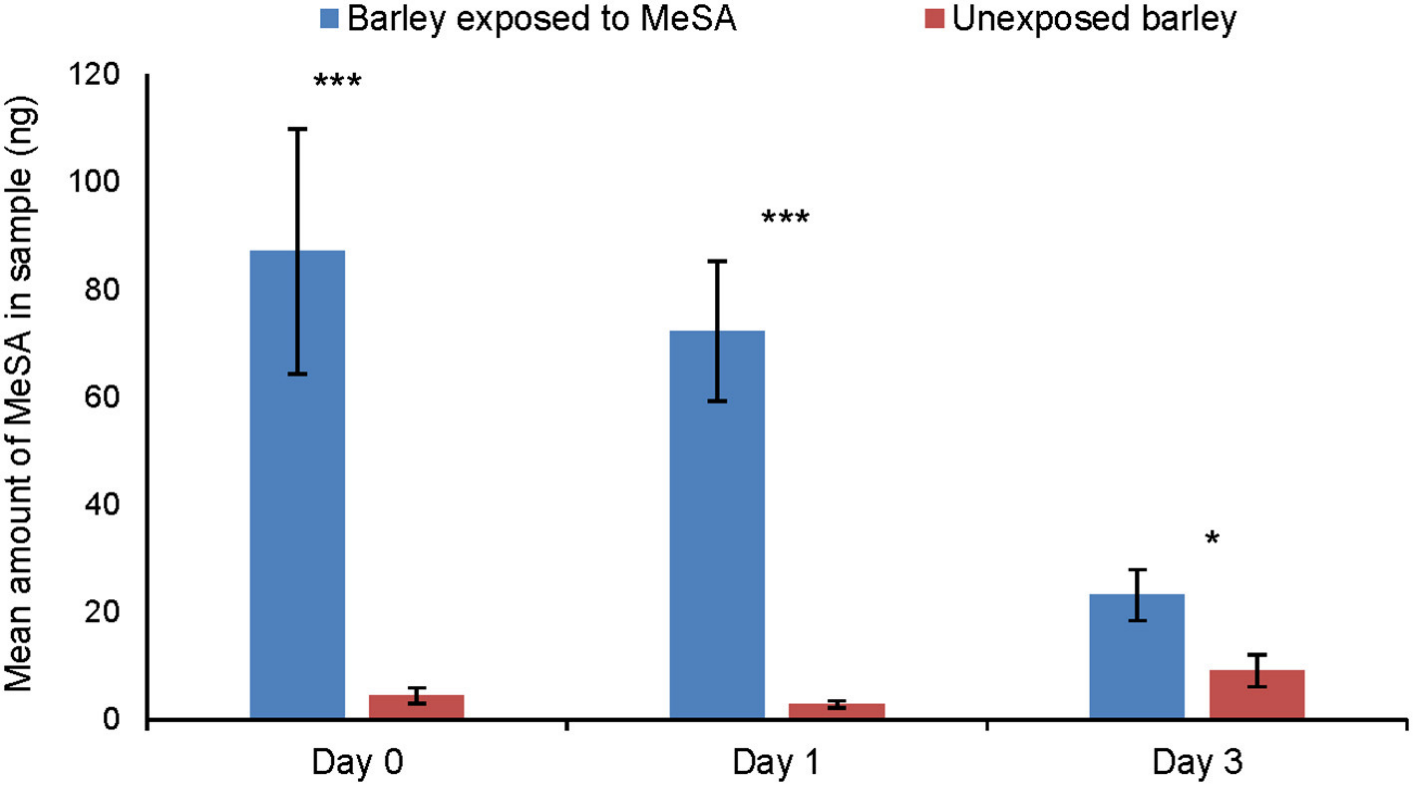
Amount of MeSA (mean ng ± SE) quantified in the headspace of unexposed barley plants or on plants exposed to MeSA (directly after removal of MeSA exposure = Day 0 and at time points 1 and 3 days after removal of MeSA exposure). *p ≤ 0.05; ***p ≤ 0.001, after Tukey test.

Significantly more methyl salicylate was detected in the headspace of MeSA exposed plants than of unexposed plants (Figure 3) at Day 0 (Tukey test, *p* = 0.0002), Day 1 (Tukey test, *p* = 0.0002) and Day 3 h (Tukey test, *p* = 0.013) after the end of exposure.

**Figure 4:**
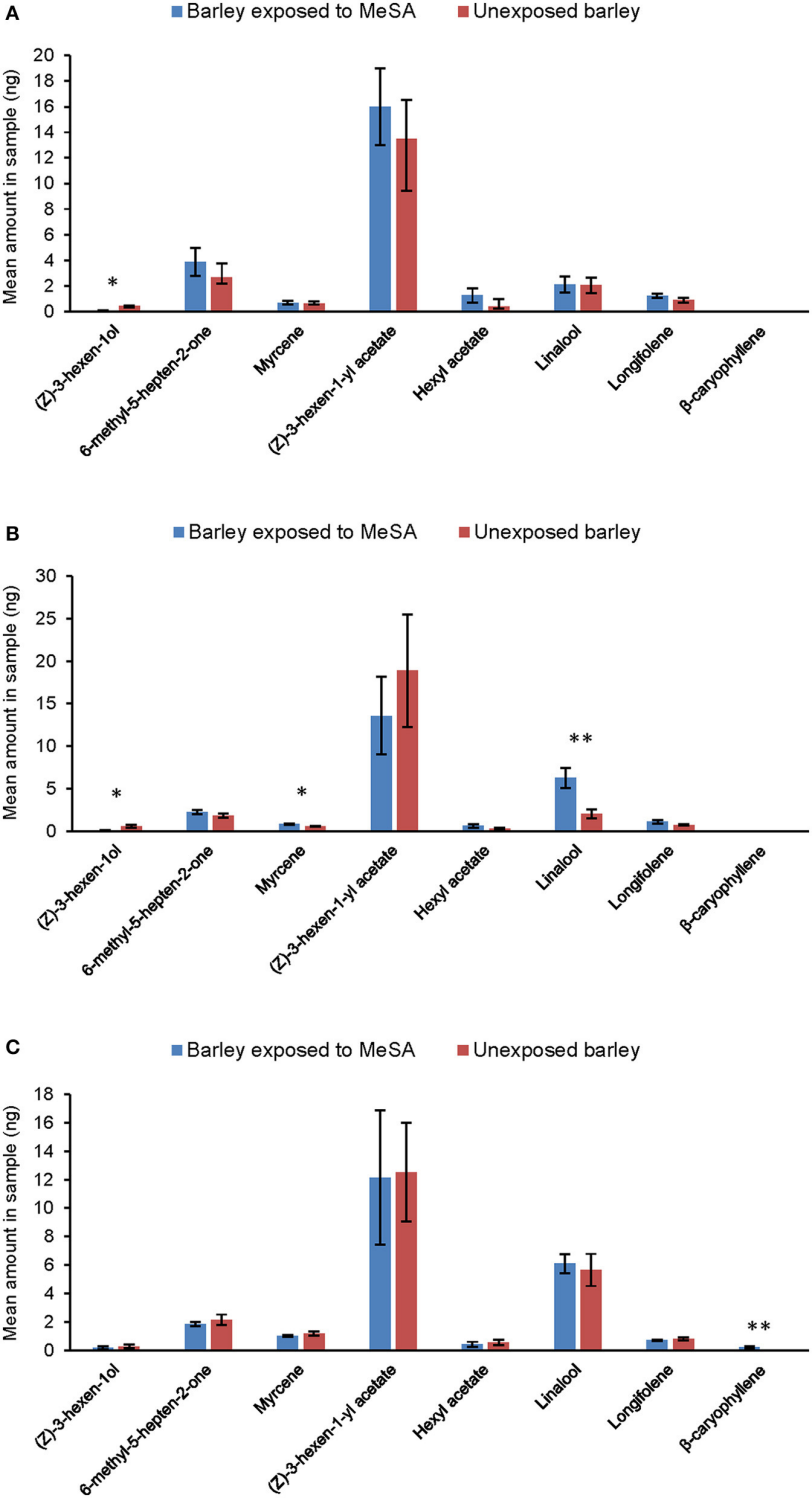
Amounts of volatile compounds (mean ng ± SE) quantified in the headspace of unexposed barley plants and plants exposed to MeSA [directly after removal of MeSA exposure (Day 0) **(A)** and at time points 1 **(B)** and 3 days **(C)** after removal]. *p ≤ 0.05; **p ≤ 0.01, after ANOVA. MeSA was analyzed in the headspace but, due to large differences in amounts, it is shown in a separate figure (Figure 3).

Exposure to methyl salicylate resulted in significant quantitative and qualitative changes in the occurrence of certain volatiles in barley headspace. At Day 0 (Figure 4A), exposed plants released significantly less of the green leaf alcohol (*Z*)-3-hexen-1-ol than unexposed plants (ANOVA, *F*_1, 10_ = 9.04, *p* = 0.013). At Day 1 (Figure 4B), exposed plants released significantly less (Z)-3-hexen-1ol than unexposed plants (ANOVA, *F*_1, 10_ = 15.17, *p* = 0.002) and significantly more of the terpenoids myrcene (ANOVA, *F*_1, 10_ = 9.33, *p* = 0.12) and linalool (ANOVA, *F*_1, 10_ = 13.8, *p* = 0.004). At Day 3 (Figure 4C), exposed plants released the sesquiterpene (*E*)-β-caryophyllene, which was not detected from unexposed plants (ANOVA, *F*_1, 10_ = 20.18, *p* = 0.001).

### Estimation of Absorption/Re-release Compared With *DeNovo* Production of MeSA in Exposed Plants

The relative abundances in the headspace of deuterated MeSA (characterized by ion *m/z* = 155) and undeuterated MeSA (*m/z*= 152) showed that a very high proportion of the methyl salicylate quantified in the headspace of exposed plants in the experiments above was most likely absorbed and re-released from the plant (98.7% at Day 0, 96.2% at Day 1, 87.7% at Day 3) (Supplementary Table 1). The amount of deuterated MeSA recorded was initially high at Day 0 and decreased on Days 1 and 3 in a similar pattern to that seen with the earlier experiment above. A higher amount of undeuterated MeSA in the headspace of exposed compared to unexposed plants would indicate induced *de novo* production, but this was significantly higher only on Day 1 (*p* ≤ 0.01 Mann-Whitney U Test).

### Aphid Olfactory Response to Synthetic Odor Blends

**Figure 5:**
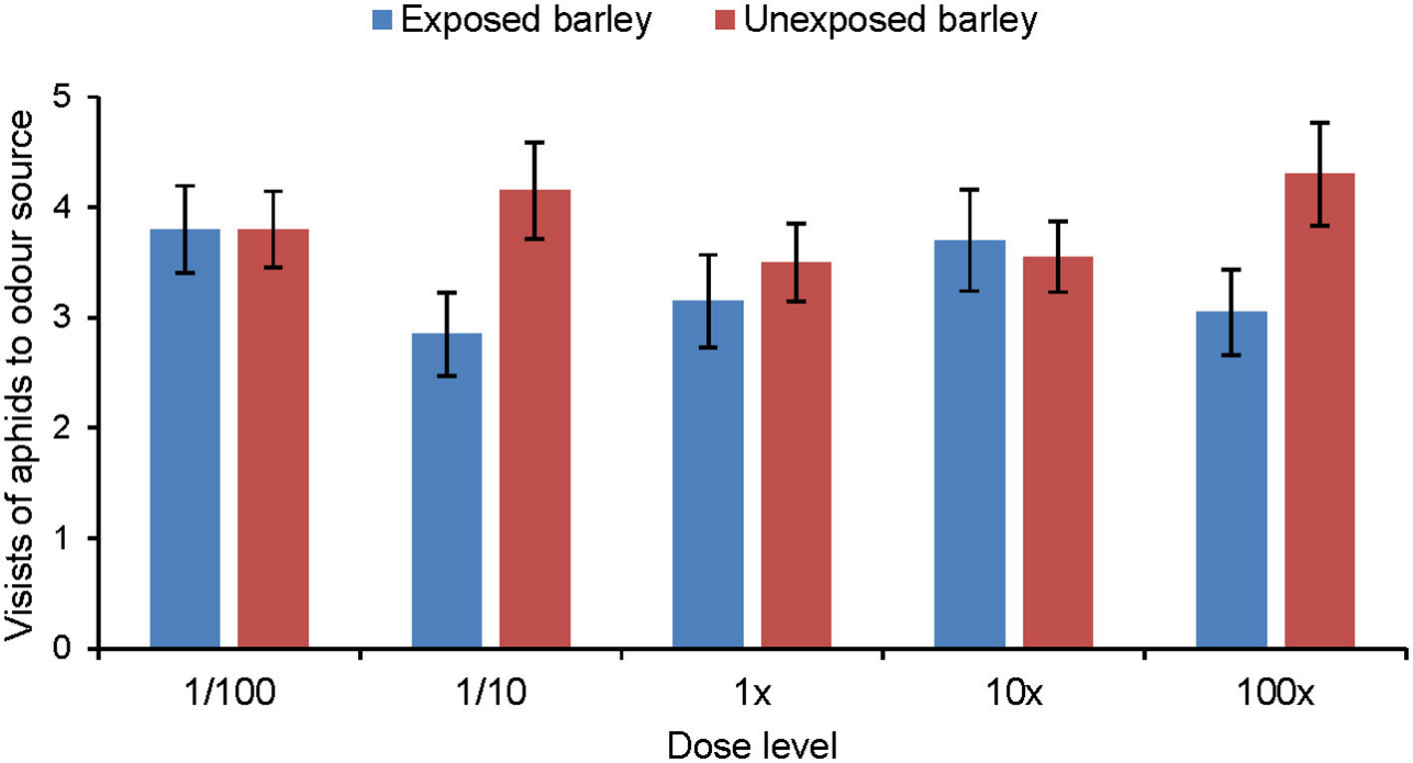
Olfactory response of *R. padi* (mean visits in olfactometer arm ± SE) to different concentrations of a synthetic volatile blend resembling the headspace of unexposed barley plants or plants exposed to methyl salicylate. (Wilcoxon matched pairs tests did not show significant differences).

**Figure 6:**
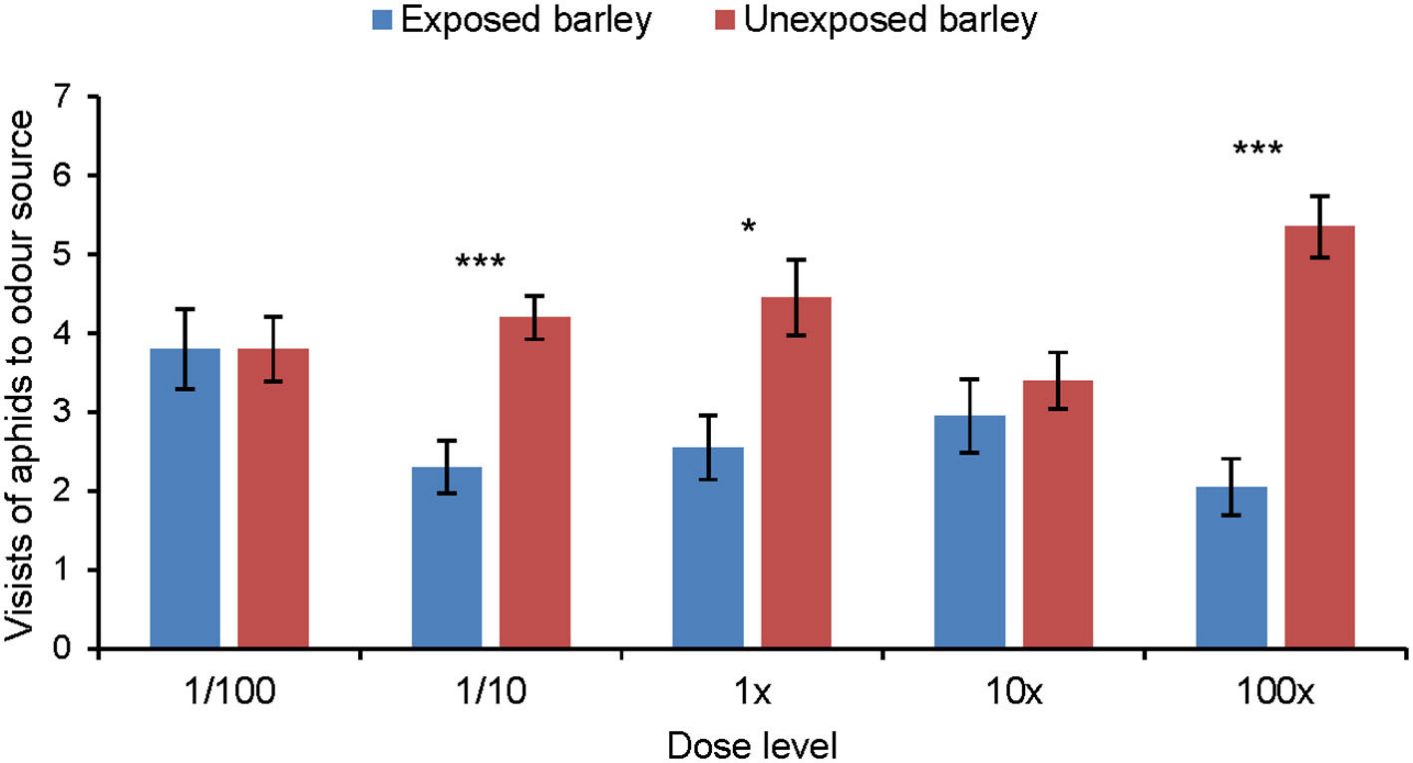
Olfactory response of *R. padi* (mean visits in olfactometer arm ± SE) to different concentrations of a synthetic volatile blend resembling the headspace of unexposed barley plants or plants exposed to methyl salicylate, but with MeSA not included in the blend. *p ≤ 0.05; ***p ≤ 0.001 after Wilcoxon matched pairs tests.

**Figure 7:**
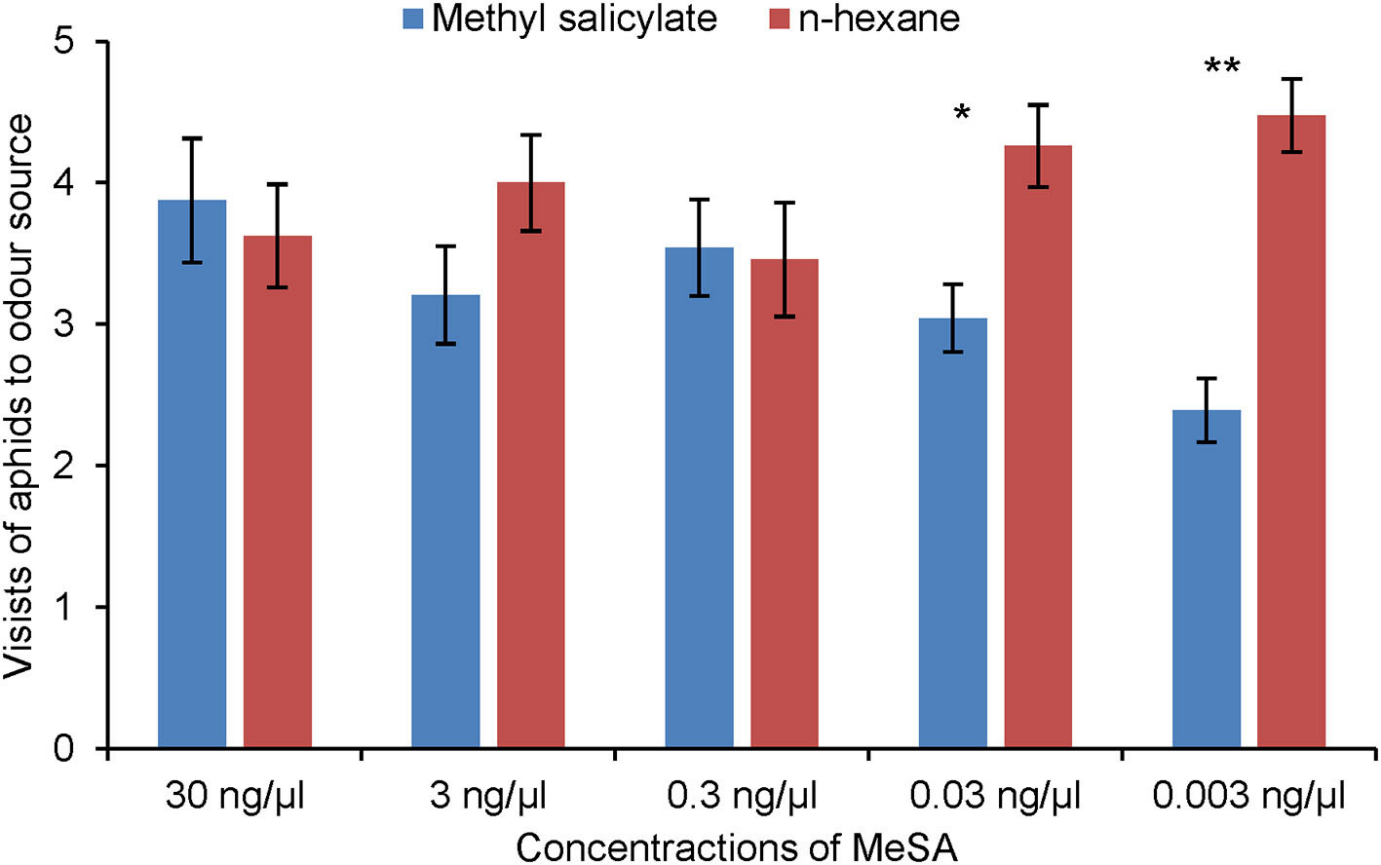
Olfactory response of *R. padi* (mean visits in olfactometer arm ± SE) to different concentrations of MeSA compared with control (hexane). *p ≤ 0.05; **p ≤ 0.01 after Wilcoxon matched pairs tests.

Aphids did not show any significant response to the synthetic blend of MeSA-exposed barley compared with the synthetic blend of unexposed barley (Figure 5). Aphids did discriminate between the synthetic blends of MeSA-exposed and unexposed barley plants when MeSA was excluded from the synthetic blends (Figure 6). Aphids made significantly fewer visits to the olfactometer arm containing the synthetic blend of MeSA-exposed barley than to the arm containing the blend of unexposed barley when solutions with concentrations of 10 ng/μl (Wilcoxon test: Z = 2.897, *p* = 0.004, *n* = 15), 1 ng/μl (Wilcoxon test: Z = 2.225, *p* = 0.026, *n* = 17) and 0.01 ng/μl (Wilcoxon test: Z = 3.416, *p* = 0.0006, *n* = 20) were used as odor sources (Figure 7).

### Aphid Olfactory Response to Individual Compounds That Differed Significantly Between Volatile Profiles of MeSA-Exposed and Unexposed Plants

Aphids made significantly fewer visits to the olfactometer arm containing MeSA than to the arm containing hexane control, but only at the two lowest concentrations tested, 0.03 ng/μl (Wilcoxon test: *Z* = 2.44, *p* = 0.015, *n* = 17) and 0.003 ng/μl (Wilcoxon test: Z = 3.07, *p* = 0.002, *n* = 22) (Figure 7). Aphids did not respond to (*Z*)-3-hexen-1-ol or linalool (Supplementary Figures 1, 2).

### Probing Behavior of Aphids on MeSA-Exposed and Unexposed Plants

Table 1 gives a summary of effects on aphid feeding, the full data are presented in Supplementary Table 2. Immediately after 24 h exposure to MeSA (Day 0), the phloem feeding time in MeSA-exposed plants was significantly shorter than in unexposed plants (*p* = 0.02) and the number of feeding periods longer than 10 and 60 mins were significantly fewer in MeSA-exposed compared to unexposed plants (*p* = 0.01; *p* = 0.001) (Supplementary Table 2A). The duration of pathway (C), which represents intercellular stylet penetration from epidermis to phloem, was not significantly different in exposed plants (*p* = 0.44). There was no significant difference in the number of short probes (<3 min) (*p* = 0.63) or the number of short probes before the 1st phloem phase (*p* = 0.72). There was no significant difference in salivation period (E1) between MeSA-exposed and unexposed plants (*p* = 0.44).

**Table 1:** Summary of effects of exposure of barley to MeSA on feeding behaviors of *R. padi* on barley plants investigated using electronic penetration graph (EPG) directly after removal of MeSA exposure and at time points 1 and 3 days after removal.

**EPG variables**	**24 h after exposure**	**Days after exposure**		
		**1 day**	**3 days**	**5 days**
**Plant surface**				
1. Time to 1st probe	–	–	–	–
2. Duration of all non-probing	–	–	++(longer)	–
**Epidermis and mesophyll**				
3. Total duration of all probes	–	–	++(shorter)	–
4. Number of probing before 1st E1	–	++(higher)	–	–
5. Total duration of all path (C)	–	++(longer)	–	–
6. Number of all path (C)	–	++(higher)	–	–
**Xylem**				
6. Duration of all G	–	++(longer)	–	–
7. Number of all G	–	++(higher)	–	–
**Phloem**				
8. Duration of 1st E12 period	++(shorter)	–	–	–
8. Duration of all E12 period	++(shorter)	–	++(shorter)	–
10. Duration of all E1 fractions	–	++(longer)	–	–
11. Duration between 1st E1 and 1st E12	–		++(longer)	–
12. Duration between 1st E1 and 1st E12 w/o non–probing	–	–	++(longer)	–
13. Duration all single E1	–	++(longer)	–	–
14. Duration of 1st E2 fractions	++(shorter)	–	–	–
15. Duration of all E2 fractions	++(shorter)	–	++(shorter)	–
16. Duration of all E	++(shorter)	–	++(shorter)	–
17. Number of all single E1	–	++(higher)	–	–
18. Number of E2 fraction more than 10 min	++(lower)	–	++(lower)	–
19. Number of E2 fraction more than 60 min	++(lower)	–	++(lower)	–
20. Duration of all E1	–	++(longer)	–	–
21. Number of all E12 periods	–	–	++(lower)	–

*Effects noted in the table with ++ indicate statistical significant differences between exposed and unexposed plants (see Results and Supplementary Table 2)*.

Twenty four hours after removal of MeSA (Day 1) the significantly longer salivation period (E1) (*p* = 0.03) and the higher number of E1 (*p* = 0.02) and duration of all E1 fractions (*p* = 0.046) in MeSA-exposed plants (Supplementary Table 2B). Mean duration of the feeding period (E2) was shorter in MeSA-exposed plants, but not significantly. Duration of probing did not differ between exposed and unexposed plants (*p* = 0.57) but in exposed plants significantly longer time was spent in the path period (about 40%) (*p* = 0.014) and less time in the feeding period (E2) (about 29%) compared to unexposed plants (26 and 50% respectively). Number of probes before the 1st feeding attempt (1st E1) was significantly higher in MeSA-exposed plants (*p* = 0.042). The duration of the xylem feeding period was significantly longer and the number of xylem feeding periods (G) was significantly higher in MeSA-exposed plants (*p* = 0.034; *p* = 0.008 respectively).

Three days after removal of MeSA (Day 3) the duration of non-probing period was significantly longer (*p* = 0.024) while probing period was shorter (*p* = 0.023) in MeSA-exposed plants than unexposed (Supplementary Table 2C). The effect was mainly due to a significantly shorter duration of feeding period (E2) in exposed plants (*p* = 0.03). The number of long sustained feeding periods (> 10 and 60 mins) was significantly higher in unexposed plants (*p* = 0.037; *p* = 0.048). A further indication of resistance to feeding in MeSA-exposed plants is the longer time between the 1st E1 and 1st E12 (sustained feeding) period (*p* = 0.013). This means that aphids took a longer time to establish a successful feeding period in exposed plants.

Five days after removal of MeSA (Day 5), the EPG results show no significant effects of plant exposure to MeSA on aphid probing (Supplementary Table 2D).

## Discussion

This study shows that exposing plants to volatile MeSA can impact the step-wise process of *R. padi* host plant selection by affecting aphid behavior at more than one step. The findings support the potential of MeSA as a compound that can protect crop plants against this aphid pest by interfering with the host selection process by different modes of action.

MeSA has been shown to act as a mobile signal for systemic acquired resistance (SAR) via reconversion to salicylic acid (SA) in the plant (Park et al., 2007). SA triggers defenses and plays a critical role in plant immunity to phloem-feeding insects, including aphids (Kaloshian and Walling, 2005; Goggin, 2007; Smith and Boyko, 2007; Spoel and Dong, 2012; van Dam et al., 2018). Exogenous application of the SA analog benzothiadiazole (BTH) induces plant defenses and has been shown to disrupt aphid colonization and feeding behavior in wheat (Cao et al., 2014) and population growth rates on susceptible and resistant tomato cultivars (Li et al., 2002; Cooper et al., 2004). In the current study we show that exposure of barley to the volatile signal MeSA negatively affects aphid host selection. We also report for the first time MeSA-induced disruption of different stages of the aphid feeding process as revealed by EPG. SA analogs tend to be less phytotoxic than SA itself (Durrant and Dong, 2004; Tripathi et al., 2019), and application of BTH reduced foliar thickness and caused necrotic lesions on sprayed tomato leaves (Boughton et al., 2006). The plants exposed to MeSA in the current study did not show any visible changes during the experiment period, but further investigation of effects on plant development and yield are needed.

The first step in host selection by aphids involves orientation to color and volatile cues from the host plant (Pettersson et al., 2007). In the olfactometer, aphids avoided the odor of MeSA-exposed plants but only 1 and 3 days after termination of the 24 h exposure to MeSA; there was no significant aphid response immediately after termination of exposure (Day 0) or 5 days after termination. Exposure to MeSA did cause significant changes in the volatile profile of barley. This suggests that MeSA can induce changes in the biochemistry of exposed plants. Compounds that were induced or upregulated by MeSA-exposure were mainly terpenoids, which are known to be involved in plant defensive responses (Mumm et al., 2008). However, it is unclear whether these induced changes in barley volatile profiles affected aphid behavior; an artificial volatile blend designed to replicate that of MeSA-exposed plants at Day 1 was less attractive to aphids than the blend of unexposed plants, but only when the blend lacked the high proportion of MeSA itself that was recorded in the headspace. Further, several of the volatiles that were altered in MeSA-exposed plants did not affect aphid orientation in the olfactometer when tested individually. Plant volatiles may have different effects on aphid behavior when encountered alone or together with other compounds in blends (Bruce and Pickett, 2011). For practical reasons, our study used wingless aphid morphs for the experiments, whereas it can be argued in nature that the initial steps in host location and selection are carried out by winged morphs.

Aphids were repelled by MeSA itself in the olfactometer, but only when it was introduced at the lower concentrations in a dose-response experiment. Thus the olfactory responses to MeSA-exposed plants observed at Days 1 and 3 could be due to a concentration-dependent response to MeSA in the headspace. Repellency of MeSA has been reported for *R. padi*, the Russian wheat aphid *Diuraphis noxia* (Glinwood and Pettersson, 2000; Prinsloo et al., 2007) and black bean aphid, *Aphis fabae* (Hardie et al., 1994). The current results suggest that the *R. padi* olfactory response to MeSA is dynamic and concentration-dependent, possibly representing an adaptation to biologically-relevant levels. Interestingly, the olfactory response of *R. padi* to MeSA has also been shown to vary dynamically within migratory aphid individuals depending on life stage (Glinwood and Pettersson, 2000). It is probable that the olfactory response of *R. padi*, a host-alternating species, to MeSA has evolved in relation to the compound's role in plant defense and the aphid's lifecycle including dispersal of apterous aphids on the summer host plant (Glinwood and Pettersson, 2000; Pickett et al., 2017).

By exposing plants to deuterated MeSA, we attempted to determine whether the high levels of MeSA in the headspace of exposed plants were most likely absorbed and re-released from the plant or produced *de novo*. While the results show some *de novo* production, they suggest that the majority of MeSA from exposed plants that was available to aphids as an olfactory cue in our experiments was absorbed then re-released from the leaves/stem. It is still unknown whether uninfested plants can adsorb and re-release MeSA produced by infested neighbors, thus gaining protection without the metabolic costs of producing the compound. The absorption and re-release of volatiles from plant tissues has been shown to affect arthropods and pathogens in several systems, and this mechanism could potentially contribute protective effects to crops in pest management (Himanen et al., 2010; Mofikoya et al., 2019; Camacho-Coronel et al., 2020). Ideally, the dynamics between re-release and *de novo* production after MeSA exposure should be studied over a longer period of plant development and in a field situation.

When aphids have contacted the host plant, they make an assessment of host suitability before settling and initiating the probing and feeding process (Pettersson et al., 2007). The settling bioassay showed that after exposure to MeSA, aphid settling was significantly reduced on exposed plants immediately following the termination of the exposure period (Day 0). A significant reduction in settling on exposed plants was also found at two subsequent time points after termination of exposure Day 1 and Day 3, but on Day 5 there was no significant difference between settling on exposed and unexposed plants. This reduction in settling could be partially explained by a response to MeSA absorbed on the leaves, and the EPG data do suggest responses to plant surface factors at Day 3. However, the settling data do not correlate fully with the aphid's olfactory responses, suggesting that plant exposure to MeSA also induced systemic resistance factors within the plant. These were investigated by the Electrical penetration graph (EPG) study of aphid probing and feeding.

EPG has been widely used to monitor aphid stylet activities on plants and to identify plant tissues where resistance factors against aphids are expressed (Tjallingii, 2006). The EPG data in the current study suggest that exposure to MeSA results in a leaf surface resistance factor, indicated by a contact effect on the initial probing behavior (a longer time from the start of the EPG recording until the first probe) of *R. padi* on exposed compared to unexposed plants at Day 3. A prolonged period before the first probe reflects the effect of repellent or deterrent surface factors (Alvarez et al., 2006). However, this effect was not statistically significant at Day 0 and Day 1, which may indicate slow induction of systemic resistance factors.

Probing and non-probing time, an indication of the suitability of the plant for feeding, was not significantly different at Day 0 suggesting the absence of induced resistance factors on plant surface and in the epidermis and mesophyll. Aphids spent less time in the phloem phase (E12) and had a shorter first sustained feeding period (E12) in exposed compared to unexposed plants. Shorter feeding times and fewer long feeding periods in exposed plants suggest that a relative short 24 h exposure to MeSA can induce changes in barley phloem sap making the plant less suitable for aphids.

After Day 1, there was no significant difference in probing time and sustained phloem feeding time, but there was a significantly longer xylem feeding time in exposed plants. It appears that resistance factors are located in both mesophyll and phloem sieve elements. Resistance in the mesophyll is suggested by the significantly longer all path period (C) and significantly higher number of probes before the first feeding attempt (1st E1) in exposed plants. Resistance in the phloem is suggested by significantly longer salivation (E1) period and lower mean feeding time (E2) in exposed plants. Cao et al. (2014) suggested that the increased salivation period is due to the fact that SA primes phloem clogging processes. Tjallingii (2006) found the salivation period (single E1 and all E1 fractions) was considerably increased in frequency and duration in resistant cultivars of melon and potato, suggesting aphids had difficulties to initiate phloem sap ingestion. Garzo et al. (2002) suggested that a prolonged E1 salivation and reduced E2 indicate a reduced ability to suppress the phloem wound response in resistant cultivars. The consequence of this is that aphids spend more time searching for a suitable feeding site through the mesophyll, and more time in combating resistance factors in the sieve elements.

Resistance factors affecting *R. padi* outside of the phloem have been reported in wild barley (a possible role of hydroxamic acids) (Niemeyer, 1990). A high gramine content has been detected in barley epidermis and mesophyll parenchyma cells, but not in phloem vessels, causing *R. padi* to take longer to reach the phloem in seedlings with high gramine levels (Zúñiga et al., 1988). There is a clear correlation between the presence of a resistance factor (hydroxamic acids) in the mesophyll and the time cereal aphids including *R. padi* take to reach the phloem. The aphids spent more time searching for a suitable phloem vessel, with increasing frequency of probes and periods of ingestion from xylem (Givovich and Niemeyer, 1995). In a study on the aphid *Sitobion fragariae* on wheat, Ramírez and Niemeyer (1999) concluded that a high concentration of hydroxamic acids is associated with a delay in the time to start salivation in the sieve elements and an increase in the process of salivation itself, suggesting that these compounds may act both in the epidermis/mesophyll and in the phloem.

Three days after treatment (Day 3) *R. padi* allocated significantly less time in probing and more to non-probing on MeSA-exposed plants, suggesting systemic induction of resistance factors. Further evidence for this is the shorter duration of phloem phase (E12) and feeding period (E2) in exposed plants. Long feeding periods (longer than 10 and 60 mins), indicating stable, sustained feeding, were also significantly reduced in exposed plants suggesting a resistance factor in the phloem.

The EPG data show that exposure of barley to MeSA can induce resistance factors against aphids that negatively affect probing even 3 days after exposure has ended. However, 5 days after exposure ended there were no significant effects on the first steps in aphid host selection.

The potential of MeSA to contribute to sustainable plant protection has been demonstrated for a number of different pests and crops, and via different mechanisms including attraction of natural enemies (James, 2003; Sasso et al., 2007, 2009; Mallinger et al., 2011; Orre Gordon et al., 2013; Rubene et al., 2019; Byers et al., 2021). Several studies have shown that releasing volatile MeSA in cereal crops can reduce aphid populations (Pettersson et al., 1994; Ninkovic et al., 2003; Prinsloo et al., 2007; Xu et al., 2018). The current study confirms this potential against aphid pests, and suggests that MeSA may disrupt aphid host selection at several stages, including olfactory orientation, settling and feeding. The EPG data suggest that exposing plants to MeSA can induce systemic resistance factors that interfere with aphid feeding. Our results support previous hypotheses that MeSA may play a multi-functional role in plant protection against aphids, contributing both with a repellent olfactory effect and an induced plant resistance effect (Pettersson et al., 1994; Ninkovic et al., 2003; Sasso et al., 2007; Digilio et al., 2012). The disruption of aphid host selection induced by MeSA disappeared 5 days after the exposure had been terminated. This suggests that to take advantage of induced plant resistance, a continuous release of MeSA in the crop during the establishment of aphid populations would be preferred over a short-lived application. In fact, the studies reporting reduction of aphid populations in crops used slow-release formulations of MeSA (Pettersson et al., 1994; Ninkovic et al., 2003; Prinsloo et al., 2007; Xu et al., 2018). Multiple modes of action could be an advantage in a pest management scenario since it may reduce the risks of aphids developing genotypes that overcome one of the modes, and offer protection against one of the modes being disrupted, for example by abiotic factors such as extreme weather.

In conclusion this study has revealed new insights into the mechanisms by which methyl salicylate can disrupt aphid host selection and supports its potential as a tool for sustainable management of aphid pests in cereals. A key question for its success will be the development of environmentally and economically sustainable application technologies.

## Data Availability Statement

The raw data supporting the conclusions of this article will be made available by the authors, without undue reservation.

## Author Contributions

VN designed the study. VN and AGÜ conducted the experiments. RG, SG, and CU conducted chemical analysis. VN analyzed the data. VN and RG wrote the manuscript. All authors read, contributed to revisions, and approved the manuscript.

## Conflict of Interest

The authors declare that the research was conducted in the absence of any commercial or financial relationships that could be construed as a potential conflict of interest.

## Publisher's Note

All claims expressed in this article are solely those of the authors and do not necessarily represent those of their affiliated organizations, or those of the publisher, the editors and the reviewers. Any product that may be evaluated in this article, or claim that may be made by its manufacturer, is not guaranteed or endorsed by the publisher.
