# Supplementary material for: Effects of Methyl Salicylate on Host Plant Acceptance and Feeding by the Aphid Rhopalosiphum padi
Source: Front Plant Sci. 2021 Aug 13;12:710268. doi: 10.3389/fpls.2021.710268 (PMC8415113; doi:10.3389/fpls.2021.710268)

SYNTHESIS OF DEUTERATED METHYL SALICYLATE (D-MeSA)

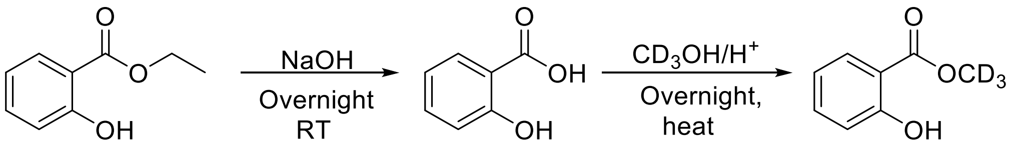

Step 1: Synthesis of salicylic acid

To a solution of ethyl salicylate (5 g, 30.1 mmol) in acetonitrile (10 mL) was added NaOH (2.4 g, 60.2 mmol) in 10 mL H_2O and stirred it for overnight at room temperature. After completion of the reaction, acetonitrile was evaporated from the reaction flask and diluted with ethylacetate. Aqueous layer was separated and acidified by treating with 2N HCl, and then extracted with ethylacetate. Organic layer washed with brine solvent and dried over $MgSO_4$ before concentrated on rotavacuum to get a pure while colour crystalline salicylic acid. Yield 70% (2.9 g, 21.1 mmol), GC-MS: R_t 8.27 *m*/*z* 120 (100%), 92, 138 (M^+), 64.

Step-2: Synthesis of deuterated methyl salicylate

A mixture of salicylic acid (500 mg, 3.6 mmol), methanol-d_4 (0.3 mL, 7.2 mmol) and a catalytic amount of conc. HCl (0.2 mL) were stirred at 50 °C heating temperature for overnight. The next day reaction mixture diluted with ethyl acetate and organic layer was separated. The organic layer was washed with brine solvent and dried over $MgSO_4$ before concentrated on rotavacuum to get a crude mixture, which was purified on silicagel column chromatography to yield 350 mg of deuterated methyl salicylate (Yield 62%, 2.3 mmol)

^1H NMR (400 MHz, $CDCl_3$) δ (in ppm) 10.76 (1H, s, -OH), 7.83 (1H, d, J = 8.0 Hz), 7.45 (1H, t, J = 7.7 Hz), 6.97 (1H, d, J = 8.4 Hz), 6.87 (1H, t-like, J = 7.5 Hz). ^{13}C NMR (100 MHz, $CDCl_3$) δ (in ppm) 170.5, 161.5, 135.6, 129.8, 119.1, 117.5, 112.3. GC-MS: R_t 7.51 *m*/*z* 120 (100%), 155 (M^+), 92, 121, 65, 93.

GC chromatograms of ethyl salicylate conversion to deuterated methyl salicylate on a non-polar capillary GC column. (DB-5MS, 30 m x 0.25 µm, ID 0.25 mm, J&W Scientific, USA)

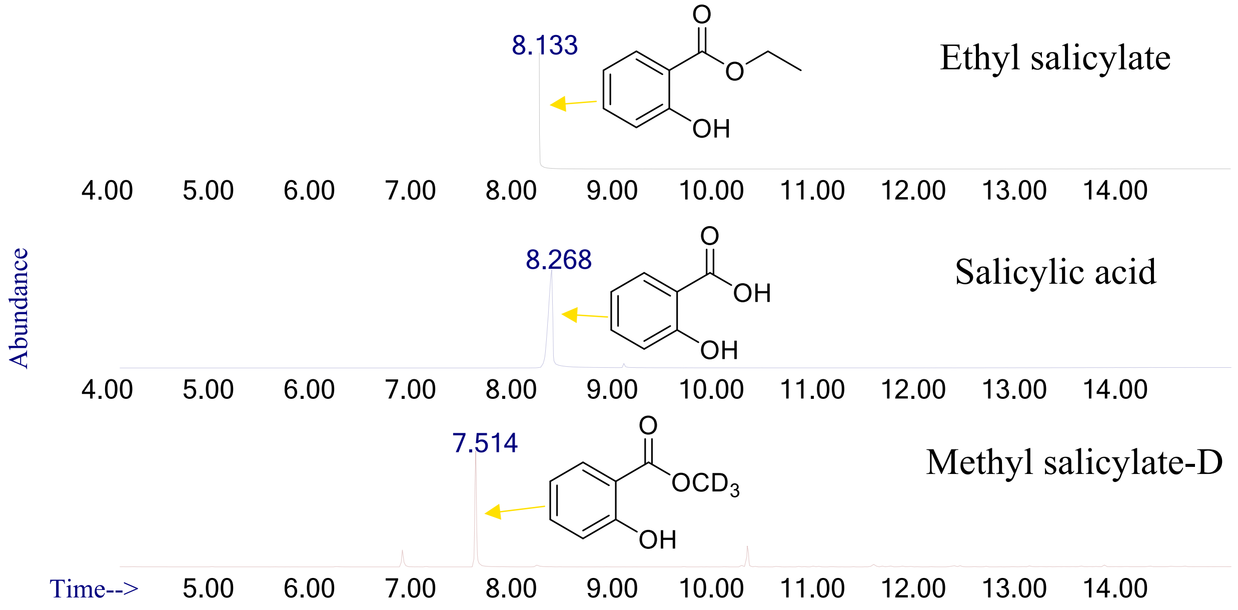

GC chromatographic separation of deuterated and non-deuterated methyl salicylate on a non-polar capillary GC column. (DB-5MS, 30 m x 0.25 µm, ID 0.25 mm, J&W Scientific, USA)

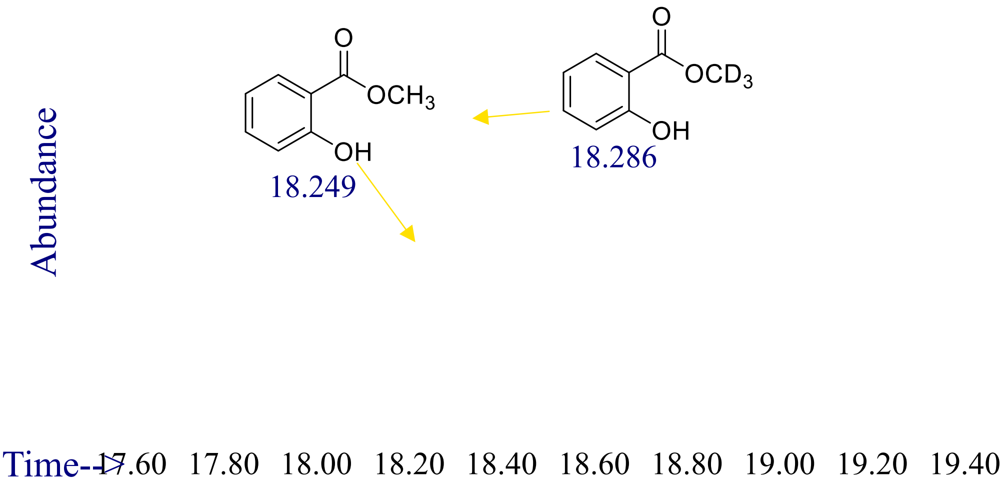

^{1}H-NMR (400 MHz, $CDCl_3$) of Deuterated methyl salicylate

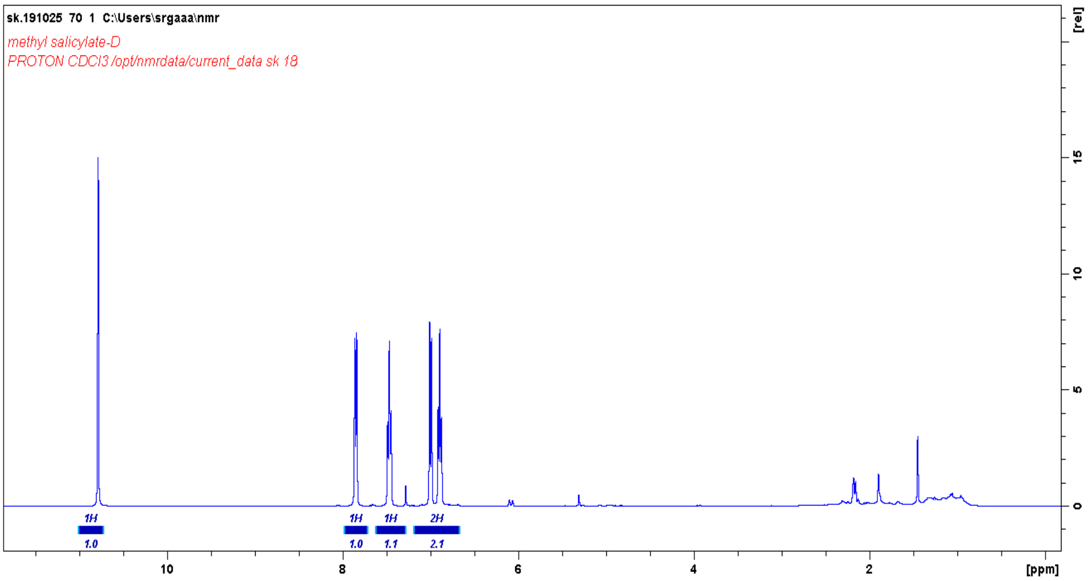

^{13}C-NMR (400 MHz, $CDCl_3$) of Deuterated methyl salicylate

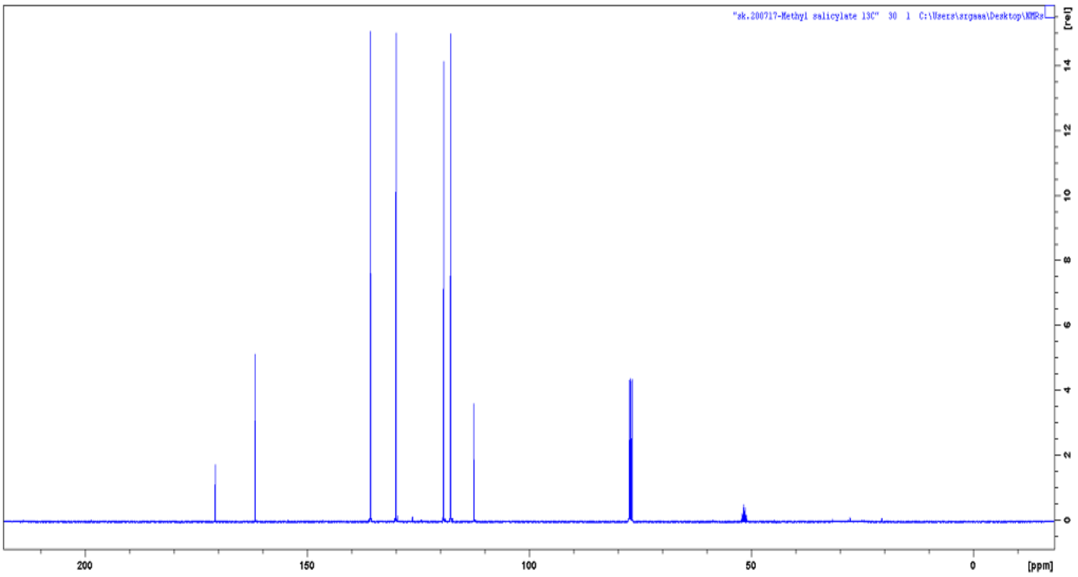

Supplement: Supplementary file 1 [file Data_Sheet_1.zip › Supplementary Material - Synthesis of Deuterated MeSA.PDF]
