# Supplementary material for: Effects of Methyl Salicylate on Host Plant Acceptance and Feeding by the Aphid Rhopalosiphum padi
Source: Front Plant Sci. 2021 Aug 13;12:710268. doi: 10.3389/fpls.2021.710268 (PMC8415113; doi:10.3389/fpls.2021.710268)
Supplement: Supplementary file 1 [file Data_Sheet_1.zip › Supplementary Table 1.PDF]

**Table S1.** Amount (mean ± se) of deuterated methyl salicylate (MeS) (quantified using ion m/z=155) and non-deuterated MeS (quantified using ion m/z=152) in the headspace of barley plants exposed to deuterated MeS for 24 hours. Volatiles collected for 24 hours starting immediately after removal of MeS (Day 0), 1 day after removal of MeS (Day 1) or 3 days after removal of MeS (Day 3). Asterisks indicate statistical significant differences between exposed and unexposed plants at * $p \leq 0.05$; ** $p \leq 0.01$ (Mann-Whitney U Test).

| **Mean amount methyl salicylate in sample (ng)** | | | | | | |
|---|---|---|---|---|---|---|
| | Day 0 | | Day 1 | | Day 3 | |
| Quant. ion | Unexposed | Exposed | Unexposed | Exposed | Unexposed | Exposed |
| *m/z*=155 | 0.10±0.04 | 28.76±10.82** | 0.00±0.0 | 4.05±0.98** | 0.08±0.07 | 1.42±0.34** |
| *m/z*=152 | 0.15±0.07 | 0.39±0.13$^{n.s.}$ | 0.02±0.02 | 0.16±0.06** | 0.05±0.04 | 0.20±0.07$^{n.s.}$ |
