# Supplementary material for: Effects of Methyl Salicylate on Host Plant Acceptance and Feeding by the Aphid Rhopalosiphum padi
Source: Front Plant Sci. 2021 Aug 13;12:710268. doi: 10.3389/fpls.2021.710268 (PMC8415113; doi:10.3389/fpls.2021.710268)
Supplement: Supplementary file 1 [file Data_Sheet_1.zip › Supplementary Table 2.PDF]

Table S2. Effects of exposure of barley to MeSA on feeding behaviours of *R. padi* on barley plants investigated using electronic penetration graph (EPG) a) directly after removal of MeSA exposure, b) at time points 1 day, c) at time points 3 days and d) at time points 5 days after removal. (p values from Mann–Whitney U-test).

Table S2a

	EPG variable	Treatment n=18	Control n=18	*p* value
	**Non probing phase**			
1	Total duration of all non probing	233.03 ± 34.62	154.53 ± 34.56	0.100
2	Number of all np	9.39 ± 1.48	10.39 ± 1.56	0.635
3	Duration of 1st np	40.01 ± 16.01	12.58 ± 3.68	0.590
4	Number of np between 1st E1 and 1st E12	1.44 ± 1.05	2.17 ± 1.28	0.752
	**Probing phase**			
5	Total duration of all probes (ABC + pd + E)*	264.05 ± 37.44	320.38 34.16	0.255
6	Number of all probes	8.94 ± 1.47	10.33 ± 1.54	0.411
7	Duration of 1st prob	70.03 ± 18.74	61.76 ± 20.78	0.164
8	Number of probes less than 3 min	4.06 ± 0.87	5.50 ± 1.34	0.621
9	Total duration of probing with 1st E1	35.66 ± 9.98	39.53 ± 9.29	0.646
10	Number of probes before 1st E1	3.00 ± 0.99	3.56 ± 0.88	0.351
11	Total duration of all path (ABC)	100.75 ± 19.88	116.26 ± 19.33	0.429
12	Number of all path (ABC)	18.28 ± 2.39	20.06 ± 2.56	0.613
	**F phase**			
13	Total duration of all F	51.05 ± 10.72	61.79 ± 10.38	0.327
14	Number of all F	4.50 ± 1.01	4.11 ± 0.92	0.899
	**G phase**			
15	Total duration of all G	38.82 ± 9.98	26.21 ± 11.23	0.242
16	Number of all G	1.11 ± 0.28	0.89 ± 0.32	0.402
	**Phloem phase**			
17	Total duration all single E1	4.33 ± 1.90	3.91 ± 1.25	0.937
18	Number of all single E1	3.06 ± 0.77	4.11 ± 1.13	0.658
19	Time to the 1st E1	106.55 ± 28.45	132.14 ± 33.32	0.517
20	Duration of 1st E12	12.83 ± 10.49	42.69 ± 20.08	**0.037**
21	Time to the 1st E12	74.86 ± 26.30	173.27 ± 37.41	**0.040**
22	Total duration of all E12 period	41.06 ± 17.13	111.90 ± 26.75	**0.025**
23	Number of all E12 periods	1.67 ± 0.55	1.78 ± 0.38	0.343
24	Duration between 1st E1 and 1st E12	12.73 ± 8.81	44.77 ± 21.82	0.496
25	Duration between 1st E1 and 1st E12 wo non probing	12.31 ± 8.58	36.09 ± 17.28	0.517
26	Total duration of all E1 fractions	9.34 ± 2.53	14.46 ± 4.68	0.438
27	Number of all E1 fractions	6.17 ± 1.68	7.06 ± 1.63	0.580
28	Duration of 1st E2 fractions	11.26 ± 9.95	39.97 ± 19.18	**0.027**
29	Total duration of all E2 fractions	36.14 ± 15.52	101.35 ± 26.04	**0.023**
30	Number of all E2 fractions	1.67 ± 0.55	1.83 ± 0.38	0.327
31	Total duration of all E	45.48 ± 16.91	115.81 ± 26.86	**0.035**
32	Number of all E	7.83 ± 2.20	8.89 ± 1.94	0.506
33	Number of E2 fractions less than 10 min	1.39 ± 0.51	0.78 ± 0.29	0.613
34	Number of E2 fractions more than 10 min	0.28 ± 0.11	1.06 ± 0.17	**0.001**
35	Number of E2 fractions more than 60 min	0.17 ± 0.09	0.50 0.12	**0.038**
36	Number of single E1 longer than 10 min	0.11 ± 0.076	0 ± 0	0.151

Table S2b

	EPG variable	Treatment n=19	Control n=19	*P* value
	Non probing phase			
1	Total duration of all non probing	159.36 ± 32.58	207.92 ± 41.38	0.511
2	Number of all np	9.95 ± 1.63	7.26 ± 1.52	0.237
3	Time of the 1st np	14.20 ± 2.88	31.19 ± 13.65	0.569
4	Number of np between 1st E1 and 1st E12	1.10 ± 1.00	0.58 ± 0.58	0.589
	Probing phase			
5	Total duration of all probes (ABC + pd + E)*	316.03 ± 32.63	270.04 ± 41.19	0.569
6	Number of all probes	9.55 ± 1.63	7.26 ± 1.50	0.307
7	Duration of 1st prob	91.82 ± 30.76	81.09 ± 35.74	0.133
8	Number of probes less than 3 min	4.75 ± 1.20	3.79 ± 0.948	0.930
9	Total duration of probing with 1st E1	34.94 ± 6.66	36.94 ± 9.92	0.474
10	Number of probes before 1st E1	4.25 1.30	2.11 ± 0.93	**0.042**
11	Total duration of all path (ABC)	125.38 ± 18.96	70.15 ± 11.94	**0.014**
12	Number of all path (ABC)	18.05 ± 2.37	11.00 ± 1.91	**0.033**
	F phase			
13	Total duration of all F	28.62 7.89	24.31 ± 11.24	0.630
14	Number of all F	2.75 ± 1.24	0.84 ± 0.36	0.373
	G phase			
15	Total duration of all G	45.02 ± 11.80	23.56 ± 8.61	**0.034**
16	Number of all G	1.45 ± 0.26	0.63 ± 0.19	**0.008**
	Phloem phase			
17	Total duration all single E1	13.71 ± 3.91	2.05 ± 1.02	**0.027**
18	Number of all single E1	4.00 ± 0.96	1.37 ± 0.52	**0.023**
19	Time to the 1st E1	133.60 ± 25.81	100.34 ± 27.99	0.157
20	Duration of 1st E12	63.65 ± 22.20	81.86 ± 34.21	0.651
21	Time to the 1st E12	123.81 ± 29.21	102.65 ± 28.74	0.550
22	Total duration of all E12 period	105.21 ± 25.99	146.85 ± 36.52	0.672
23	Number of all E12 periods	1.35 ± 0.49	1.68 ± 0.41	0.530
24	Duration between 1st E1 and 1st E12	15.49 ± 10.04	3.96 ± 2.74	0.559
25	Duration between 1st E1 and 1st E12 wo non probing	15.08 ± 10.00	3.96 ± 2.74	0.579
26	Total duration of all E1 fractions	28.10 ± 6.30	14.67 ± 5.26	**0.046**
27	Number of all E1 fractions	6.40 ± 1.62	4.21 ± 1.16	0.137
28	Duration of 1st E2 fractions	49.01 ± 19.64	68.59 ± 33.16	0.693
29	Total duration of all E2 fractions	91.22 ± 22.44	134.23 ± 35.99	0.782
30	Number of all E2 fractions	1.50 ± 0.50	1.79 ± 0.42	0.550
31	Total duration of all E	119.33 ± 25.53	148.91 ± 36.55	0.895
32	Number of all E	7.90 ± 2.04	6.00 ± 1.56	0.321
33	Number of E2 fractions less than 10 min	0.85 ± 0.45	0.89 ± 0.26	0.569
34	Number of E2 fractions more than 10 min	0.65 ± 0.13	0.89 ± 0.20	0.589
35	Number of E2 fractions more than 60 min	0.50 ± 0.11	0.47 ± 0.12	0.782
36	Number of single E1 longer than 10 min	0.30 ± 0.11	0.05 ± 0.05	0.166

Table S2c

	EPG variable	Treatment n=19	Control n=19	*P* value
	**Non probing phase**			
1	Total duration of all non probing	207.19 ± 30.04	140.85 ± 26.84	**0.024**
2	Number of all np	9.92 ± 1.23	9.41 ± 1.43	0.725
3	Duration of 1st np	31.46 ± 15.63	17.65 ± 5.57	0.660
4	Number of np between 1st E1 and 1st E12	0.83 ± 0.51	0.07 ± 0.07	0.448
	**Probing phase**			
5	Total duration of all probes (ABC + pd + E)*	271.92 ± 29.84	338.01 ± 26.94	**0.023**
6	Number of all probes	9.63 ± 1.19	9.37 ± 1.42	0.792
7	Duration of 1st prob	53.44 ± 18.19	47.44 ± 17.08	0.621
8	Number of probes less than 3 min	5.29 ± 1.01	5.22 ± 1.28	0.956
9	Total duration of probing with 1st E1	27.19 ± 6.66	36.89 ± 10.04	0.272
10	Number of probes before 1st E1	3.83 ± 1.12	3.74 ± 0.80	0.323
11	Total duration of all path (ABC)	77.74 ± 11.39	77.90 ± 10.57	0.904
12	Number of all path (ABC)	15.67 ± 1.74	15.48 ± 1.83	1.000
	**F phase**			
13	Total duration of all F	38.24 ± 7.10	38.96 ± 8.88	0.956
14	Number of all F	3.54 ± 0.94	2.37 ± 0.42	0.939
	**G phase**			
15	Total duration of all G	27.48 ± 6.63	30.49 ± 5.69	0.312
16	Number of all G	1.08 ± 0.28	1.85 ± 0.40	0.083
	**Phloem phase**			
17	Total duration all single E1	2.64 ± 0.73	2.92 ± 1.25	0.392
18	Number of all single E1	1.46 ± 0.33	1.44 ± 0.41	0.629
19	Time to the 1st E1	91.76 ± 24.73	111.72 ± 21.98	0.083
20	Duration of 1st E12	89.36 ± 27.00	113.40 ± 30.73	0.423
21	Time to the 1st E12	113.03 ± 27.62	111.46 ± 22.38	0.386
22	Total duration of all E12 period	127.18 ± 30.88	189.15 ± 32.42	**0.036**
23	Number of all E12 periods	1.04 ± 0.21	1.70 ± 0.27	**0.021**
24	Duration between 1st E1 and 1st E12	20.95 ± 11.31	5.89 ± 3.94	0.085
25	Duration between 1st E1 and 1st E12 wo non probing	18.33 ± 9.70	4.74 ± 3.22	0.085
26	Total duration of all E1 fractions	10.37 ± 2.20	7.64 ± 2.19	0.676
27	Number of all E1 fractions	3.21 ± 0.65	4.26 ± 0.85	0.258
28	Duration of 1st E2 fractions	76.71 ± 26.61	98.93 ± 29.59	0.328
29	Total duration of all E2 fractions	119.02 ± 29.97	184.16 ± 32.32	**0.030**
30	Number of all E2 fractions	1.21 ± 0.25	1.81 ± 0.29	**0.036**
31	Total duration of all E	129.39 ± 30.74	191.80 ± 32.03	**0.033**
32	Number of all E	4.42 ± 0.87	6.07 ± 1.11	0.163
33	Number of E2 fractions less than 10 min	0.58 ± 0.20	0.85 ± 0.23	0.297
34	Number of E2 fractions more than 10 min	0.63 ± 0.12	0.96 ± 0.15	**0.037**
35	Number of E2 fractions more than 60 min	0.42 ± 0.10	0.67 ± 0.12	**0.048**
36	Number of single E1 longer than 10 min	0.04 ± 0.04	0.07 ± 0.05	0.801

Table S2d

	EPG variable	Treatment n=21	Control n=21	P value
	**Non probing phase**			
1	Total duration of all non probing	163.41 ± 21.45	122.78 ± 19.50	0.222
2	Number of all np	10.24 ± 1.34	10.57 ± 1.35	0.782
3	Duration of 1st np	22.85 ± 8.08	19.19 ± 8.86	0.821
4	Number of np between 1st E1 and 1st E12	0.86 ± 0.41	0.24 ± 0.17	0.538
	**Probing phase**			
5	Total duration of all probes (ABC + pd + E)*	316.59 ± 21.45	371.45 ± 16.33	0.099
6	Number of all probes	9.86 ± 1.35	10.43 ± 1.33	0.651
7	Duration of 1st prob	42.35 ± 17.00	72.18 ± 22.78	0.333
8	Number of probes less than 3 min	4.90 ± 1.04	4.62 ± 1.05	0.930
9	Total duration of probing with 1st E1	42.31 ± 13.51	18.26 ± 4.15	0.320
10	Number of probes before 1st E1	2.43 ± 0.49	2.86 0.71	0.980
11	Total duration of all path (ABC)	115.62 ± 16.22	125.26 ± 14.67	0.660
12	Number of all path (ABC)	17.86 ± 2.16	19.76 ± 2.30	0.554
	**F phase**			
13	Total duration of all F	51.68 ± 11.25	56.84 ± 15.42	0.831
14	Number of all F	2.67 ± 0.53	3.71 ± 1.12	0.930
	**G phase**			
15	Total duration of all G	83.90 ± 20.45	56.07 ± 10.28	0.624
16	Number of all G	1.29 ± 0.22	1.67 ± 0.28	0.406
	**Phloem phase**			
17	Total duration all single E1	15.85 ± 7.42	6.39 ± 2.27	0.435
18	Number of all single E1	3.29 ± 0.94	2.76 ± 0.90	0.725
19	Time to the 1st E1	89.05 ± 23.80	96.42 ± 21.54	0.697
20	Duration of 1st E12	38.02 ± 14.16	81.60 ± 31.16	0.571
21	Time to the 1st E12	104.82 ± 27.67	100.85 ± 27.53	0.990
22	Total duration of all E12 period	49.54 ± 14.59	112.65 ± 30.75	0.252
23	Number of all E12 periods	1.29 ± 0.38	1.81 ± 0.51	0.571
24	Duration between 1st E1 and 1st E12	25.96 ± 11.68	19.41 ± 9.85	0.633
25	Duration between 1st E1 and 1st E12 wo non probing	18.37 ± 8.47	16.60 ± 8.54	0.633
26	Total duration of all E1 fractions	25.15 ± 8.35	10.48 ± 3.34	0.385
27	Number of all E1 fractions	5.62 ± 1.62	6.10 ± 1.72	0.687
28	Duration of 1st E2 fractions	25.44 ± 12.66	79.10 ± 30.86	0.346
29	Total duration of all E2 fractions	40.24 ± 13.61	108.57 ± 30.49	0.222
30	Number of all E2 fractions	1.29 ± 0.38	1.81 ± 0.51	0.571
31	Total duration of all E	65.39 ± 15.63	119.05 ± 30.52	0.359
32	Number of all E	6.90 ± 1.99	7.90 ± 2.20	0.633
33	Number of E2 fractions less than 10 min	0.62 ± 0.23	0.90 ± 0.31	0.571
34	Number of E2 fractions more than 10 min	0.67 ± 0.20	0.90 ± 0.24	0.481
35	Number of E2 fractions more than 60 min	0.19 ± 0.09	0.48 ± 0.13	0.170
36	Number of single E1 longer than 10 min	0.24 ± 0.10	0.14 ± 0.08	0.597
