# Supplementary figures and images for: Effects of Methyl Salicylate on Host Plant Acceptance and Feeding by the Aphid Rhopalosiphum padi
Source: Front Plant Sci. 2021 Aug 13;12:710268. doi: 10.3389/fpls.2021.710268 (PMC8415113; doi:10.3389/fpls.2021.710268)

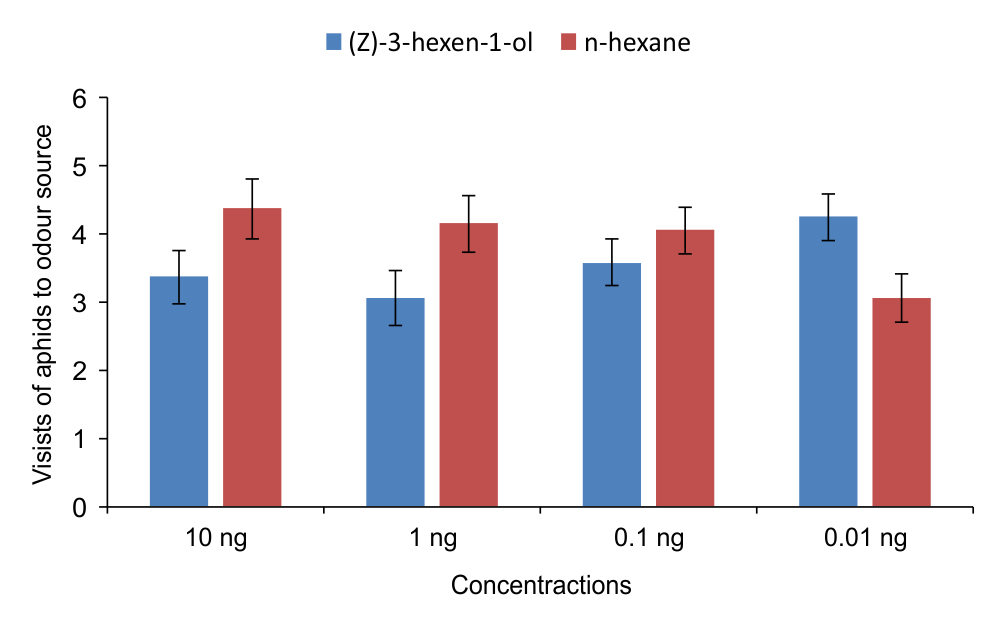

Supplement: Supplementary file 1 [file Data_Sheet_1.zip › Supplementary Figure 1.TIF]

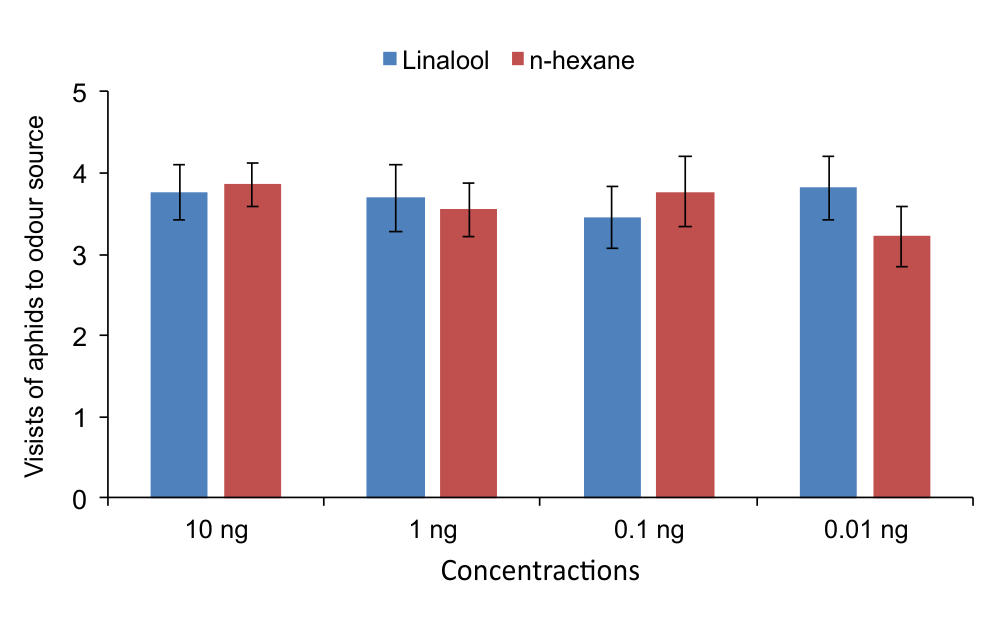

Supplement: Supplementary file 1 [file Data_Sheet_1.zip › Supplementary Figure 2.TIF]
